# Big data in healthcare: are we close to it?

**DOI:** 10.5935/0103-507X.20160008

**Published:** 2016

**Authors:** Elliot Naidus, Leo Anthony Celi

**Affiliations:** 1 Beth Israel Deaconess Medical Center - Massachussetts, United States.

## INTRODUCTION

Translating medical research into clinical practice guidelines is not trivial. There has been a surge in the number of published biomedical articles,^(1)^ but how clinicians adapt these articles into practice is not straightforward. In addition, the validity of biomedical research has recently been under scrutiny.^(2)^ Bias in publication with emphasis on sensational discoveries over reproducibility, non-acceptance of negative studies, and the academic pressure to publish have all contributed to the unreliability of biomedical research. One consequence is the "medical pendulum" phenomenon, which pertains to treatments or diagnostic tools considered beneficial one decade and later proven to be of no value, or worse, harmful. An example in critical care is the pulmonary artery catheter, which was widely adopted in the 1980s and early 1990s, but later losing favor after retrospective observational studies suggested no benefit and possible harm,^(3)^ followed by prospective randomized trials confirming such finding.^(4,5)^ And while clinical trials are best in inferring causality, they are not adept at demonstrating small effect size which is typical with most critical care intervention administered to a heterogeneous group of patients. Moreover, clinical trials typically exclude important subgroups (older patients, those with comorbidities): findings may not be generalizable to the real-world.

Because of the limitations of clinical trials including cost, many guidelines are supported by low-quality evidence.^(6)^ A survey of the American College of Obstetricians and Gynecologists practice bulletins showed only 29% of recommendations were level A, "based on good and consistent scientific evidence"^(7)^ while an appraisal of the clinical practice guidelines from the American College of Cardiology and American Heart Association found only 314 of 2,711 recommendations (11%) were based on high quality evidence.^(8)^

To make matters worse, these guidelines are often adopted in low- and middle-income countries (LMICs), including Brazil, where funding for research is limited.

Digitalization of healthcare data may provide an opportunity to develop locally relevant practice guidelines in LMICs rather than adopting those from other countries. Digital data is proliferating in diverse forms within the healthcare field, not only because of the adoption of electronic health records, but also because of the growing use of wireless technologies for ambulatory monitoring. Since clinical trials may be too expensive to perform in LMICs to inform practice guidelines, digital health data provides an opportunity to conduct locally relevant research. Rigorous observational studies have been shown to correlate well with clinical trials across the medical literature in terms of estimates of risk and effect size.^(9-11)^

### Big data as solution

Conceptually, "Big Data" includes data sets that are so large as to be considered unmanageable for human interpretation without the help of computerized data processing and/or analytics. While a challenge to traditional statistical techniques because of the level of granularity and resolution, Big Data calls for novel causal inference methodologies to model time-varying exposures and covariates. One of the use cases of Big Data in medicine is the application of machine learning techniques to predict the likelihood of events based on continuous data streams. Google, for example, employs an automated method for analyzing influenza related web searches to track the movement of the epidemic. While Google's data correlate highly with Center for Disease Control (CDC) case statistics, its method has a lead-time advantage due to analysis in real time, demonstrating a possibly better mechanism to predict and track epidemics.^(12)^ In Sierra Leone at the height of the Ebola epidemic, mobile technology was leveraged to collect large amounts of data in the villages. Real-time data analytics assisted with the quarantine efforts leading to containment of the epidemic.^(13)^

The era of Big Data and next generation analytics is well upon us. Both large data sets as well as the relevant machine learning techniques have been available for years, but they are only slowly making their way in the domain of clinical medicine.

### Big data as problem

Tyler Vigen famously published a book of spurious correlations, relating disparate trends such as the divorce rate in Maine with per capita consumption of margarine, and US spending on science, space and technology and suicides by hanging, strangulation and suffocation.^(14)^ Big Data, when analyzed without a deep understanding of the context, runs the risk of producing "big noise". The importance of cross-validation of findings, both internally and externally using other data sets, to ascertain reproducibility and evaluate for generalizability cannot be over-emphasized. Making data sets accessible to outside investigators and fostering a collaborative research ecosystem will hopefully help address the conundrum of unreliable research.

## CONCLUSION

Digitalization of health data is becoming a global phenomenon as computers, sensors and wireless technology become more prevalent. Observational studies have been shown to produce effect and risk estimates that correlate well with clinical trials. Big Data offers an opportunity for LMICs to build their own knowledge base from which to develop, continuously evaluate, and improve clinical practice guidelines specific to their populations. New causal inference methodologies may improve the field of observational studies further. To avoid the pitfalls of making "big noise" out of Big Data, it is essential to transform the process of research to be more open, self-critical and collaborative.
